# The Use of the Electro-Magnetic Mallet

**Published:** 1889-04

**Authors:** L. L. Davis


### The Use of the Electro-Magnetic Mallet.

BY L. L. DAVIS, D.D.S.

Read before the Detroit Dental Society, Monday evening March 11th, 1888.

OBSERVE—COMPARE—REFLECT—RECORD.

Four more suggestive words could not well be grouped and applied to the pursuance of any science; and when that science requires for its successful outcome an adept in manipulative skill the words assume still greater significance and importance.

Dentists are supposed to be familiar with, and competent to palliate, and if within the range of possibility, repair all lesions of the oral cavity, but unfortunately for the great mass of humanity who are obliged at one time or another to look for relief from present pains and anticipated troubles, a great majority of those who should be skilled in the means of relief and restoration to health and usefulness of the parts affected, fall far short of the sufferer's preconceived ideas of what a dentist should be.

The qualifications that are requisite for the successful prosecution of our specialty in all its branches, and the knowledge of how do deal with the conditions that are continually being presented are not, and perhaps can not be attained by all, but if our patients are dismissed with imperfectly made fillings, all of the theoretical and manipulative skill displayed in other directions will not avail to save us from condemnation and merited loss of confidence.

The territory within which we labor is circumscribed by nearly adjacent boundaries, but the injuries we are expected to correct and repair are numerous and often complicated, and demand for their successful treatment a measure of information and experience not easily acquired by every one.

"All roads lead to Rome," and all, or nearly all, of the efforts bestowed by the dentist, as such, upon the cases brought to his notice are but preparatory steps to the final and principal branch of his specialty—that of so making fillings for the carious teeth intrusted to his care that decay will seldom if ever make further progress within the cavities operated upon.

The limit of this paper does not intend or permit the discussion of all means of arresting decay in teeth, but simply of one way—that of impacting cohesive gold within the cavities by the blow given with the electro-magnetic mallet.

By way of parenthesis, however, it might be asserted at this point that if more fillings were made by the reliable, faithful, and tireless electro-magnetic mallet upon an instrument guided by the brains and fingers of conscientious and skillful operators there would be less demand for other kinds of fillings and subsequent artificial dentures.

It is now about eight years since the writer began the use of the electro-magnetic mallet and the results attained by it are so satisfactory, that of all his operations he least desires to examine are the approximal fillings in bicuspids and molars made previous to its adoption. Every operator knows that the most inconvenient place to reach and the one really difficult point to adapt gold to, is the cervical boundary of cavities in bicuspids and molars, and no one of the older operators whose inborn conceit has been lessened by repeated instances of failure in that location of his efforts, but will confess that it is with fear and trembling he examines his old cases at that point for possible and almost probable breaking down of tooth structure. Many of these failures could have been obviated, and the percentage of future liabilities in similar operations may be materially reduced by the adoption of the electro-magnetic mallet for use at that very vulnerable point of secondary decay. For no matter if every particle of decay is removed from the walls of a cavity should the gold fail to be adapted perfectly to its entire margin, secondary caries will surely occur. No argument should be needed to convince any operator of ten years experience that the regulation automatic mallet is a comparative failure as a gold adapting instrument at the point under especial consideration, for even if the serrated end can be kept within the cavity and never allowed to slip outside and wound the adjacent gum, it is hardly possible for the most painstaking operator to so place the gold as to feel entirely certain of the perfect adaptability of the filling at that point with the very limited view obtainable in cases of this kind. With these few words in regard to the use of the automatic mallet at this surface the tool is laid aside for the present and the electro-magnetic mallet is taken up to adapt the gold to the particular wall now receiving attention. Take for example one of the most difficult of all cases, a distal cavity in a superior second or third molar. None of us will have to look back many leaves of our memory to remember the inverted position the upper third of our bodies had to assume in order to obtain any view whatever of the upper half of the cavity. After the thin layer of gold or layer of tin with thin covering of gold has been placed on and across the cervical wall and secured in retaining grooves with a moderately wide but thin pointed hand-plugger, the electro-magnetic mallet and mouth mirror are brought into use and with but a slight inclination of the body a more perfect adaptation of filling to tooth can be consummated in a minute of time, than by any other known method in ten minutes by the most skilled and sure-handed operator. Continuing the operation; the gold is placed within and against the walls more rapidly, thoroughly, and satisfactorily than by any other accepted method, and showing as the case proceeds toward completion a more evenly condensed and compact mass of gold, acquired with less fatigue and annoyance to both patient and operator than by the employment of any other impacting instrument.

It is a waste of words to recall to the recollection of the operator accustomed to the use of a hand mallet, either when aimed by himself or an assistant, of the many causes that influence the number, directness, and varied quality of the blows delivered at the end of a plugging instrument. The unreliability of the assistant, and the close proximity of the patient's garments or body to the end of the instrument, are but a few of the causes of unsatisfactory malleting and consequently imperfectly constructed fillings.

The blow from the electro-magnetic mallet is always direct, always emphatic, always ready for deliverance, and always reliable when properly influenced by a correctly constructed primary battery, or by the power derived from an incandescent circuit. That a filling made by the electro-magnetic mallet is a thoroughly impacted mass of metal can be accurately tested by the use of the burnisher which leaves the surface free from those knolls, hillocks, and depressions almost always seen upon the face of a filling made by a hand or automatic mallet and burnished in a similar manner.

As an additional test of the imperfectness of the density of a filling made by the blows of a hand or automatic mallet after the surface has been thoroughly and systematically gone over with the usual points used in such cases, the electro-magnetic mallet is applied and leaves the surface of the supposed compact mass uneaven and hilly, thus demonstrating conclusively that the character of the blows delivered by the mallets first named were not sufficiently regular to make an evenly impacted filling.

The use of the electro-magnetic mallet for adapting the gold to the margins of a cavity is so superior in point of correctness and thoroughness and freedom from that liability of marring and comminution of tooth substance from illtimed and misdirected blows of other mallets that in the matter of excellence and superiority of results it is far in advance of any other tool for a similar purpose.

No valid reasons can be urged against the blow of the electro-magnetic mallet, for where the opinion and judgment of an operator requires the use of cohesive gold malleted into fillings for the salvation of teeth, nothing else so productive of satisfactory results can be used with such ease and certainty.

Many more words might be said in its favor as the scope of its usefulness is not half covered in this paper, but I will close this part of the subject by quoting the language of one now gone from us, than whom there was none superior as a maker of gold fillings for the preservation of carious teeth. In speaking of the rise and fall of the hammer on the instrument, he said, "The successive and rapid recurrence of these movements, with a full current of electricity in a properly adjusted electro-magnetic mallet enables an operator with careful and intelligent guidance of the instrument to go over the whole surface of the foil much better and make the gold more solid and uniform in density, with greater ease and rapidity than by any other known method."

The dentist's first duty is to his patients. When the results attained by his honest efforts are satisfactory to himself the case may be dismissed with confidence that the future will bring no reproach upon his skill. The dentist also owes a duty to himself —that of making his labor as free from fatigue and perplexities as possible.

Who that has stood at the chair for hours but leaves it at night thoroughly tired and exhausted from the unnatural and inverted position the body had to assume in order to see every portion of the cavity being operated upon by the hand or automatic mallet. "Tired nature's sweet restorer—balmy sleep," will cease, after a time, to bring relief to the over-taxed brain and muscles, thus defying her law, and sooner or later a demand will be made for absolute rest, or change of occupation.

The possession and use of the electro-magnetic mallet makes the operation of filling cavities with gold so easy and fascinating that the duties of the day are looked forward to with pleasure and freedom from dread.

The words of Dr. Marshall H. Webb were fitly chosen when he wrote upon this subject in this manner, "Of such assistance has it been to the writer since the spring of 1873, that he feels it has already done much toward prolonging his life."

